# Global Dissemination of Carbapenemase-Producing *Klebsiella pneumoniae*: Epidemiology, Genetic Context, Treatment Options, and Detection Methods

**DOI:** 10.3389/fmicb.2016.00895

**Published:** 2016-06-13

**Authors:** Chang-Ro Lee, Jung Hun Lee, Kwang Seung Park, Young Bae Kim, Byeong Chul Jeong, Sang Hee Lee

**Affiliations:** ^1^National Leading Research Laboratory of Drug Resistance Proteomics, Department of Biological Sciences, Myongji UniversityYongin, South Korea; ^2^Division of STEM, North Shore Community College, DanversMA, USA

**Keywords:** carbapenemase, *Klebsiella pneumoniae*, epidemiology, KPC, NDM, OXA-48-like

## Abstract

The emergence of carbapenem-resistant Gram-negative pathogens poses a serious threat to public health worldwide. In particular, the increasing prevalence of carbapenem-resistant *Klebsiella pneumoniae* is a major source of concern. *K. pneumoniae* carbapenemases (KPCs) and carbapenemases of the oxacillinase-48 (OXA-48) type have been reported worldwide. New Delhi metallo-β-lactamase (NDM) carbapenemases were originally identified in Sweden in 2008 and have spread worldwide rapidly. In this review, we summarize the epidemiology of *K. pneumoniae* producing three carbapenemases (KPCs, NDMs, and OXA-48-like). Although the prevalence of each resistant strain varies geographically, *K. pneumoniae* producing KPCs, NDMs, and OXA-48-like carbapenemases have become rapidly disseminated. In addition, we used recently published molecular and genetic studies to analyze the mechanisms by which these three carbapenemases, and major *K. pneumoniae* clones, such as ST258 and ST11, have become globally prevalent. Because carbapenemase-producing *K. pneumoniae* are often resistant to most β-lactam antibiotics and many other non-β-lactam molecules, the therapeutic options available to treat infection with these strains are limited to colistin, polymyxin B, fosfomycin, tigecycline, and selected aminoglycosides. Although, combination therapy has been recommended for the treatment of severe carbapenemase-producing *K. pneumoniae* infections, the clinical evidence for this strategy is currently limited, and more accurate randomized controlled trials will be required to establish the most effective treatment regimen. Moreover, because rapid and accurate identification of the carbapenemase type found in *K. pneumoniae* may be difficult to achieve through phenotypic antibiotic susceptibility tests, novel molecular detection techniques are currently being developed.

## Introduction

The increasing prevalence of antibiotic resistance and the lack of new antibiotic drug development has gradually reduced the treatment options for bacterial infections (Lee et al., 2013a; Nathan and Cars, 2014). In 2013, the Centers for Disease Control and Prevention (CDC) named three microorganisms that pose an urgent threat to public health: carbapenem-resistant (CR) *Enterobacteriaceae* (CRE), drug-resistant *Neisseria gonorrhoeae*, and *Clostridium difficile* (Zowawi et al., 2015). Carbapenems (imipenem, meropenem, biapenem, ertapenem, and doripenem) are antibiotics used for the treatment of severe infections caused by multi-resistant *Enterobacteriaceae*, such as *Klebsiella pneumoniae* and *Escherichia coli* (Nordmann et al., 2009). However, over the past 10 years, CRE have increasingly been reported worldwide (Nordmann et al., 2011a). In particular, *K. pneumoniae* have acquired carbapenemases, which are enzymes capable of breaking down most β-lactams including carbapenems, and thus conferring resistance to these drugs (Jeon et al., 2015). High mortality rates have been reported in patients with bloodstream infections caused by CR *K. pneumoniae* (Munoz-Price et al., 2013). Carbapenemases can be divided according to their dependency on divalent cations for enzyme activation into metallo-carbapenemases (zinc-dependent class B) and non-metallo-carbapenemases (zinc-independent classes A, C, and D; Jeon et al., 2015). The class A carbapenemases, such as the *K. pneumoniae* carbapenemase (KPC) enzymes, have been identified worldwide in *K. pneumoniae* (Tangden and Giske, 2015). Various class B and D carbapenemases have also been detected in hospital-acquired multi-resistant *K. pneumoniae* (Nordmann et al., 2011a), whereas class C carbapenemases have rarely been reported. In this review, we summarize the epidemiology of the major four classes of carbapenemases and discuss their molecular genetics, methods used for their detection, and the therapeutic options available for their treatment.

## The Epidemiology, Genetic Context, Treatment Options, and Detection Methods of Carbapenem-Resistant *K. pneumoniae*

### Class A Carbapenemases

#### Epidemiology

Various class A carbapenemases forming six distantly related branches have been identified (Jeon et al., 2015). While some carbapenemases are chromosome-encoded (IMI-1, NMC-A, SME enzymes, SHV-38, and SFC-1), others are plasmid-encoded (KPC enzymes, GES enzymes, and IMI-2). KPCs have been the most frequently observed class A carbapenemases since their first description in the eastern the USA in 1996 (Yigit et al., 2001). Of the many different KPC family variants (KPC-1 to KPC-22), the most well-characterized variants are KPC-2 and KPC-3. KPCs are mostly plasmid-encoded enzymes and bacteria producing these enzymes are susceptible to only a few antibiotics such as colistin, aminoglycosides, and tigecycline. Therefore, the mortality of the patient’s bloodstream infections caused by these bacteria is very high (Munoz-Price et al., 2013).

**FIGURE 1:**
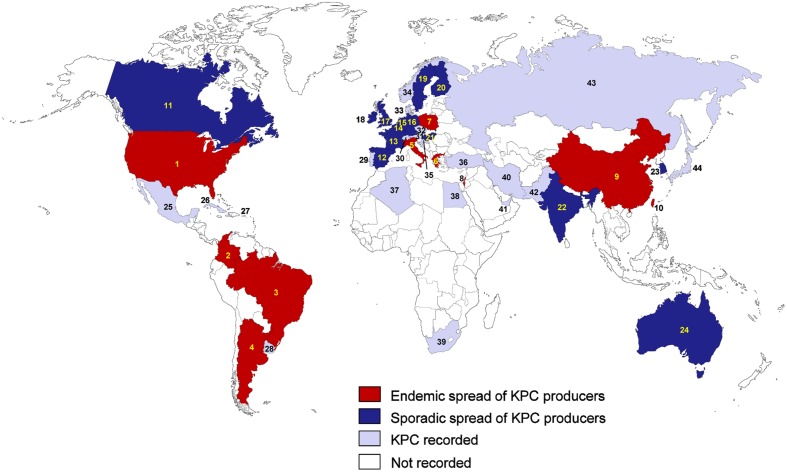
**Epidemiological features of KPC-producing *Klebsiella pneumoniae*.** (1) USA; (2) Colombia; (3) Brazil; (4) Argentina; (5) Italy; (6) Greece; (7) Poland; (8) Israel; (9) China; (10) Taiwan; (11) Canada; (12) Spain; (13) France; (14) Belgium; (15) Netherlands; (16) Germany; (17) UK; (18) Ireland; (19) Sweden; (20) Finland; (21) Hungary; (22) India; (23) South Korea; (24) Australia; (25) Mexico; (26) Cuba; (27) Puerto Rico; (28) Uruguay; (29) Portugal; (30) Switzerland; (31) Austria; (32) Czech Republic; (33) Denmark; (34) Norway; (35) Croatia; (36) Turkey; (37) Algeria; (38) Egypt; (39) South Africa; (40) Iran; (41) United Arab Emirates; (42) Pakistan; (43) Russia; (44) Japan.

The epidemiology of *K. pneumoniae* producing KPCs varies geographically. The endemic spread of these bacteria has been reported in the USA, China, Italy, Poland, Greece, Israel, Brazil, Argentina, Colombia, and Taiwan (Munoz-Price et al., 2013; **Figure 1**). Sporadic spread of KPC-producing *K. pneumoniae* has also been observed in many European countries including Spain, France, Germany, the Netherlands, the UK, Ireland, Belgium, Sweden, and Finland, and in several countries in the Asia-Pacific region, including India, South Korea, and Australia (Munoz-Price et al., 2013; Nordmann and Poirel, 2014). In the USA, the transmission of CR *K. pneumoniae* is primarily driven by the spread of organisms carrying KPC enzymes (Kaiser et al., 2013), but other carbapenemase enzymes, such as the New-Delhi metallo-β-lactamase (NDM), have also emerged (Lascols et al., 2013). Within the USA, the prevalence of KPC-positive isolates was relatively stable between 2007 and 2009 (5.9% in 2007, 4.9% in 2008, and 5.7% in 2009; Kaiser et al., 2013), and KPC-2 and KPC-3 were the most frequently identified carbapenemases in *K. pneumoniae* (Deshpande et al., 2006).

The outbreaks caused by KPC-producing *K. pneumoniae* have been reported in the USA (Woodford et al., 2004) and Israel (Leavitt et al., 2007), but recently, similar outbreaks associated with patients traveling to endemic areas have also been reported in many European counties. Since KPC-producing *K. pneumoniae* was identified in France, Italy, and Greece (Naas et al., 2005; Tsakris et al., 2008; Giani et al., 2009), the sporadic spread of KPC-producing *K. pneumoniae* has been observed in many European countries including Spain (Robustillo Rodela et al., 2012), France (Carbonne et al., 2010), Germany (Wendt et al., 2010; Steinmann et al., 2011), the Netherlands (Weterings et al., 2015), the UK (Woodford et al., 2008; Virgincar et al., 2011), Ireland (Roche et al., 2009; Morris et al., 2012), Belgium (Bogaerts et al., 2010), Sweden (Samuelsen et al., 2009), and Finland (Osterblad et al., 2012; Kanerva et al., 2015). KPC-producing *K. pneumoniae* were also recently detected in eastern European countries including the Czech Republic (Hrabak et al., 2013b), Hungary (Toth et al., 2010), and Croatia (Bedenic et al., 2012).

In Greece, KPC-producing *K. pneumoniae* was first isolated in August 2007 (Tsakris et al., 2008), and the prevalence of KPC-producers among carbapenemase-producing *K. pneumoniae* isolates collected at a tertiary Greek hospital increased from 0% in 2003 to 38.3% in 2010 (Zagorianou et al., 2012). Most of the genotyped KPC-producing *K. pneumoniae* in Greece harbored KPC-2 (Zagorianou et al., 2012). While many carbapenemase-producing *K. pneumoniae* in the USA and Greece had KPC enzymes (Nordmann et al., 2009; Zagorianou et al., 2012), several studies in Spain showed that most carbapenemase-producing *K. pneumoniae* harbored OXA-48-like or class B carbapenemases, and the rate of KPC- producing *K. pneumoniae* was very low (2–3%; Oteo et al., 2013b; Palacios-Baena et al., 2016). These results indicate that the prevalent genotype of carbapenemase-producing *K. pneumoniae* varies geographically. For example, in Italy which is a representative southern European country where KPC is becoming endemic, 89.5% of carbapenemase producers have been reported to have KPC-type enzymes, followed by VIM-1 (9.2%) and OXA-48 (1.3%; Giani et al., 2013).

In America, the endemic spread of KPCs has been reported in Colombia (Villegas et al., 2006; Rojas et al., 2013), Brazil (Peirano et al., 2009; Fehlberg et al., 2012), and Argentina (Pasteran et al., 2008; Gomez et al., 2011). In Canada, KPC-producing *K. pneumoniae* has sporadically been reported (Goldfarb et al., 2009; Lefebvre et al., 2015), and since plasmid-mediated KPC-producing *K. pneumoniae* was first detected in Ottawa in Goldfarb et al. (2009), a laboratory surveillance program found a high frequency (89.3%) of KPC-type enzymes among carbapenemase producers between 2010 and 2012 (Lefebvre et al., 2015). The emergence of KPCs in Argentina was characterized by two patterns of dispersion: the first was the irregular occurrence of diverse *Enterobacteriaceae* harboring *bla*_KPC-2_ in the IncL/M transferable plasmid in distant regions and the second was the sudden clonal spread of *K. pneumoniae* ST258 harboring *bla*_KPC-2_ in Tn*4401a* (Gomez et al., 2011). KPC-producing *K. pneumoniae* was recently also detected in Cuba (Quinones et al., 2014), Mexico (Garza-Ramos et al., 2014), Uruguay (Marquez et al., 2014), and Puerto Rico (Gregory et al., 2010).

In the Asia-Pacific region, the endemic dissemination of KPC-producing *K. pneumoniae* has been reported in China (Li et al., 2014) and Taiwan (Tseng et al., 2015), and the sporadic spread has been reported in India (Shanmugam et al., 2013), South Korea (Yoo et al., 2013), and Australia (Partridge et al., 2015). A novel KPC-15 variant which is closely homologous with KPC-4 was discovered in China (Wang et al., 2014b) and its enzymatic activity and phenotype was characterized (Wang et al., 2014a). In China, the frequency of KPC-type enzymes among carbapenemase producers was high (63%; Li et al., 2014). While ST258 is the predominant clone observed in European countries and the USA (Giani et al., 2013; Chen et al., 2014e; Bowers et al., 2015), ST11, which is closely related to ST258, is the prevalent clone associated with the spread of KPC-producing *K. pneumoniae* in Asia (particularly in China and Taiwan; Qi et al., 2011; Yang et al., 2013; Tseng et al., 2015). KPC-producing ST11 strain has also been reported in Latin America (Munoz-Price et al., 2013). Although it is unknown why ST11 is prevalent, a recent report showed that the KPC-producing *K. pneumoniae* ST11 clone was resistant to serum killing (Chiang et al., 2016). In a Chinese hospital, another nosocomial outbreak of KPC-2-producing *K. pneumoniae* was caused by multiple *K. pneumoniae* strains including ST37, ST392, ST395, and ST11, implying the horizontal transfer of *bla*_KPC-2_ gene between different *K. pneumoniae* clones in China (Yang et al., 2013). In Taiwan, two novel KPC variants were identified; KPC-16 and KPC-17 differed from KPC-2 by two (P202S and F207L) and a single (F207L) amino acid substitutions, respectively (Yu et al., 2015). A nationwide survey in Taiwan between 2011 and 2013 reported the national spread of KPC-2 and KPC-17 (Tseng et al., 2015). KPC-producing *K. pneumoniae* was recently also detected in Japan (Saito et al., 2014), Pakistan (Pesesky et al., 2015), Iran (Nobari et al., 2014), and United Arab Emirates (Sonnevend et al., 2015a). In the Arabian Peninsula, the prevalence of KPC-producing *K. pneumoniae* was very low in comparison to NDM-1 and OXA-48-like carbapenemases (Sonnevend et al., 2015b). Sonnevend et al. (2015a), two *K. pneumoniae* ST14 strains producing KPC-2 were first identified in the United Arab Emirates of the Arabian Peninsula. In Africa, several countries such as South Africa (Brink et al., 2012), Algeria (Bakour et al., 2015b), and Egypt (Metwally et al., 2013), have also isolated KPC-producing *K. pneumoniae*.

The coexistence of KPCs and other carbapenemases in *K. pneumoniae* was frequently reported worldwide, including in Italy (KPC-3/VIM-2 and KPC-2/VIM-1; Richter et al., 2012; Perilli et al., 2013), Colombia (KPC-2/VIM-24; Rojas et al., 2013), Brazil (KPC-2/NDM-1; Pereira et al., 2015), China (KPC-2/NDM-1, KPC-2/CMY-2, and KPC-2/IMP-4; Hu et al., 2014; Dong et al., 2015; Liu et al., 2015), Canada (KPC-3/CMY-2; Leung et al., 2012), and Greece (KPC-2/VIM-1; Giakkoupi et al., 2009), indicating the worldwide prevalence of *K. pneumoniae* co-harboring two carbapenemases.

Aside from KPC-type carbapenemases, other class A carbapenemases, such as GES-2, GES-4, GES-5, GES-6, GES-11, GES-14, GES-18, SFC-1, SHV-38, NMC-A, SME-1, and IMI-type enzymes, were rarely found in *K. pneumoniae* (**Table 1**).

**Table 1:** The epidemiology of various carbapenemases in *Klebsiella pneumoniae*.

Molecular class	Carbapenemase	Geographical distribution
A	SME types	Not found
	IMI types	Not found
	GES types	Greece (Vourli et al., 2004), Finland (Osterblad et al., 2012), Brazil (Picao et al., 2010), and South Korea (Jeong et al., 2005; Bae et al., 2007)
	SFC-1, SHV-38, and NMC-A	France (Poirel et al., 2003) and Brazil (Tollentino et al., 2011)
B	OXA-23, OXA-24/40, OXA-51, OXA-58, OXA-134, OXA-143, OXA-211, OXA-213, OXA-214, OXA-229, and OXA-235	Not found
C	DHA-1	Taiwan (Lee et al., 2012b), South Korea (Park et al., 2013), and China (Hu et al., 2014)
	CMY-2 and CMY-10	China (Hu et al., 2014), Canada (Leung et al., 2012), and Greece (Pournaras et al., 2010b)
	ADC-68	Not found
D	IMP types	Malaysia (Hamzan et al., 2015), Taiwan (Tseng et al., 2015), China (Chen et al., 2015), Thailand (Rimrang et al., 2012), Ireland (Morris et al., 2016), Greece (Lascols et al., 2013), Spain (Lascols et al., 2013), Italy (Lascols et al., 2013), Turkey (Lascols et al., 2013), Austria (Zarfel et al., 2011), the USA (Limbago et al., 2011; Rojas et al., 2013), and Mexico (Gales et al., 2012)
	VIM types	Greece (Pournaras et al., 2010b), Ireland (Morris et al., 2016), Spain (Pena et al., 2014), Australia (Lascols et al., 2013), Croatia (Zujic Atalic et al., 2014), the Czech Republic (Hrabak et al., 2013b), Hungary (Melegh et al., 2014), Italy (Giani et al., 2013), Norway (Naseer et al., 2012), Austria (Zarfel et al., 2011), Finland (Osterblad et al., 2012), Germany (Steinmann et al., 2011), France (Birgy et al., 2011), China (Liu et al., 2015), India (Castanheira et al., 2011), Philippines (Lascols et al., 2013), Iran (Rajabnia et al., 2015), Taiwan (Tseng et al., 2015), Colombia (Rojas et al., 2013), Mexico (Gales et al., 2012), and Algeria (Rodriguez-Martinez et al., 2010)
	GIM-1, KHM-1, and SPM-1	Not found


#### Molecular and Genetic Context

**FIGURE 2:**
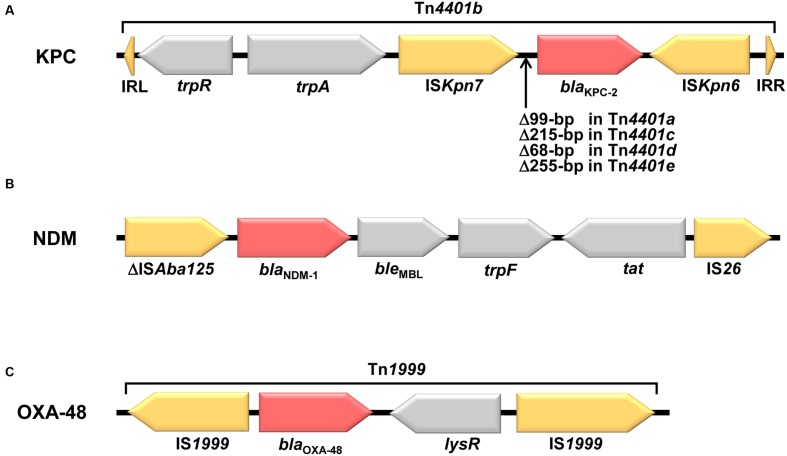
**Structural features of representative genetic environments of *bla*_KPC-2_, *bla*_NDM-1_, and *bla*_OXA-48_ genes.** **(A)** The *bla*_KPC-2_-containing Tn*4401* transposon from the plasmid pNYC (GenBank accession no. EU176011) is shown in horizontal arrows. Two inverted repeat sequences (IRL and IRR) of Tn*4401* are depicted in triangles at either end. Tn*4401* has five isoforms which differ by deletions (68–255 bp) just upstream of the *bla*_KPC_ gene [(a) deletion of 99 bp; (b) no deletion; (c) deletion of 215 bp; (d) deletion of 68 bp; (e) deletion of 255 bp]. **(B)** The *bla*_NDM-1_ genetic context of pNDM_MGR194 (GenBank accession no. KF220657) is shown in horizontal arrows. **(C)** The *bla*_OXA-48_-containing Tn*1999* transposon from the plasmid pOXA-48 (GenBank accession no. JN626286) is shown in horizontal arrows.

The *bla*_KPC_ in *K. pneumoniae* has been reported on numerous plasmid types, such as IncF, IncI2, IncX, IncA/C, IncR, and ColE1 (Garcia-Fernandez et al., 2012; Chen et al., 2014e; Pitout et al., 2015), but the predominant plasmid type is IncF with FII_K_ replicons (Pitout et al., 2015). IncF often contains several additional genes responsible for resistance to other antibiotics, including aminoglycosides, tetracyclines, quinolones, trimethoprim, and sulfonamides (Pitout et al., 2015). Many *bla*_KPC_ genes are associated with a promiscuous transposon-related structure Tn*4401*, which is approximately 10 kb in size and consists of a transposase gene, a resolvase gene, the *bla*_KPC_ gene, and two insertion sequences, IS*Kpn6* and IS*Kpn7* (**Figure 2A**; Naas et al., 2008). This transposon has jumped to numerous plasmids that are commonly conjugative (Chen et al., 2014e). In China, a novel genetic environment was detected (Shen et al., 2009). It contains an integration structure consisting of a Tn*3*-based transposon and partial Tn*4401* segment, with the gene order Tn*3*-transposase, Tn*3*-resolvase, IS*Kpn8*, the *bla*_KPC-2_ gene, and the IS*Kpn6*-like element (Shen et al., 2009). This genetic structure is the chimera form of several transposon-associated elements. This transposon was also identified in many other countries (Chen et al., 2014e), and several variants with various fragment insertions between the IS*Kpn8* and *bla*_KPC_ gene have been found among *Enterobacteriaceae* in China (Shen et al., 2009; Li et al., 2011; Qi et al., 2011). Tn*4401* has five isoforms which differ by deletions (68–255 bp) just upstream of the *bla*_KPC_ gene [(a) deletion of 99 bp; (b) no deletion; (c) deletion of 215 bp; (d) deletion of 68 bp; (e) deletion of 255 bp; Chen et al., 2014e]. Notably, in many cases, different Tn*4401* isoforms was associated with different *bla*_KPC_-harboring plasmids. Tn*4401a* was frequently found in the *bla*_KPC-3_-harboring IncFII_K2_ plasmids (Leavitt et al., 2010; Garcia-Fernandez et al., 2012; Chen et al., 2014d), and Tn*4401b* and Tn*4401d* were often associated with the IncN and IncFIA plasmids, respectively (Chen et al., 2013a, 2014c,e). Up to now, more than 30 *bla*_KPC_-harboring plasmids obtained from *K. pneumoniae* have been sequenced (Gootz et al., 2009; Shen et al., 2009; Jiang et al., 2010; Leavitt et al., 2010; Almeida et al., 2012; Chen et al., 2013a,b, 2014b,c,e). One of common features shared by these sequenced plasmids is the presence of the *tra* operon, which encodes the plasmid conjugation machinery proteins that induce the spread of plasmids (Chen et al., 2014e). These genes may be important for the successful dissemination of *bla*_KPC_-harboring plasmids. The IncFII plasmids are one of predominant *bla*_KPC_-harboring plasmids. pKpQIL, which is an IncFII_K2_ plasmid harboring Tn*4401a*, was initially identified in Israel in 2006 (Leavitt et al., 2010), and then this plasmid and its variants are believed to have spread to Italy, Poland, the UK, Colombia, the Czech Republic, the USA, and other countries (Baraniak et al., 2011; Garcia-Fernandez et al., 2012; Hidalgo-Grass et al., 2012; Warburg et al., 2012; Hrabak et al., 2013b; Chen et al., 2014d,e), suggesting the wide dissemination of this plasmid. The *bla*_KPC_ gene has also been identified in other non-Tn*4401* mobile elements that mostly have partial IS*Kpn6* genes (Shen et al., 2009; Gomez et al., 2011). Based on the insertion sequence upstream of the *bla*_KPC_ gene, they can be divided into three groups: group I, no insertion (Shen et al., 2009; Liu et al., 2012; Chen et al., 2014g); group II, insertion of truncated *bla*_TEM_ (Gomez et al., 2011); group III, insertion of Tn*5563*/IS*6100* (Wolter et al., 2009). These non-Tn*4401* genetic elements harboring *bla*_KPC_ sometimes have an IS*26* transposon (Liu et al., 2012; Chen et al., 2014g).

Carbapenemase genes often spread worldwide through clonal expansion in several successful pathogenic strains (Chen et al., 2014e). For example, the dissemination of KPC-producing *K. pneumoniae* in most countries including the USA and European countries is largely caused by expansion of a single dominant strain, ST258 (Giani et al., 2013; Chen et al., 2014e; Bowers et al., 2015). This strain is a prototype of a high-risk clone of *K. pneumoniae*. Recent data from Israel showed that the KPC-producing *K. pneumoniae* ST258 clone remains the predominant clone, representing 80% of the KPC-producing *K. pneumoniae* population (Adler et al., 2015). ST258 may be a hybrid clone that was created by a large recombination event between ST11 and ST442 (Pitout et al., 2015). It is unknown why the ST258 lineage is the most prevalent clone of KPC-producing *Klebsiella* species. The ST258 clone is highly susceptible to serum killing in animal models and lacks well-known *K. pneumoniae* virulence factors, such as aerobactin genes, K1, K2, and K5 capsular antigen genes, and the regulator of the mucoid phenotype gene *rmpA* (Tzouvelekis et al., 2013; Pitout et al., 2015). Two recent reports revealed that the ST258 strains consist of two distinct genetic clades and genetic differentiation between the two clades (*-1* and *cps-2*) results from an approximately 215-kb region of divergence that includes *cps* genes involved in capsule polysaccharide synthesis (Chen et al., 2014e; Deleo et al., 2014). Multiplex PCR for the identification of two capsular types in *K. pneumoniae* ST258 strains revealed a significant association between the *cps* type and KPC variant: the *cps-1* clade is largely associated with KPC-2, while the *cps-2* clade is primarily associated with KPC-3 (Chen et al., 2014a). Because the capsule polysaccharide can help *K. pneumoniae* to evade phagocytosis, the global success of this strain may involve the capsule polysaccharide biosynthesis regions *cps-1* and *cps-2*. A recent report revealed a relationship between the integrative conjugative element ICEKp258.2 and the global success of the ST258 clone (Adler et al., 2014). ICEKp258.2 contains two specific gene clusters, a type IV pilus gene cluster (i.e., *pilV*) associated with the uptake and exchange of plasmids and adherence to living and non-living surfaces, and a gene cluster of a type III restriction-modification system determining host specificity in the exchange of certain compatible plasmids or mobile elements (Adler et al., 2014). Because these genes associated with the restriction of plasmids and specific mobile elements were present only in ST258 and genetically related sequence types, this difference may explain the divergence of ST258 predominantly harboring KPC and ST11, another high-risk clone that lacks ICEKp258.2, harboring a broad range of plasmids and carbapenemases, including KPC, NDM, OXA-48, VIM, and IMP (Chen et al., 2014f; Pitout et al., 2015). Although the ICEKp258.2 of ST258 strains may contribute to global success, the precise reason for the predominance of the ST258 strain in KPC-producing *K. pneumoniae* is still not entirely understood. Recently, an outbreak of non-ST258 KPC-producing *K. pneumoniae* clones has been reported in the USA and Europe (Ruiz-Garbajosa et al., 2013; Bonura et al., 2015; Garbari et al., 2015).

The habitat of *K. pneumoniae* is not limited to humans but extends to the ecological environment, such as soil, water, and sewage, and *K. pneumoniae* can survive in extreme environments for long periods of time (Pitout et al., 2015). Therefore, *K. pneumoniae* producing KPCs were detected in various nosocomial environments, such as gowns and gloves (Rock et al., 2014) and wastewater (Chagas et al., 2011; Galler et al., 2014). The frequency of KPC-producing *K. pneumoniae* contamination of gowns and gloves of healthcare workers is similar to that of contamination with methicillin-resistant *Staphylococcus aureus* and vancomycin-resistant *Enterococcus* (Rock et al., 2014), indicating fast transmission of KPC-producing *Klebsiella* species in a nosocomial environment. A long-term observation in a hospital with low-frequency outbreaks of KPC-producing *K. pneumoniae* suggested the possible role of a persisting environmental reservoir of resistant strains in the maintenance of this long-term outbreak (Tofteland et al., 2013). After discharge from the hospital, long-term (>3 years) carriage of KPC-producing *K. pneumoniae* is also possible (Lübbert et al., 2014), and lateral gene transfer of KPC among *Enterobacteriaceae* colonizing the human intestine appears frequent, for example from *K. pneumoniae* to *E. coli* (Richter et al., 2011; Gona et al., 2014). Therefore, reservoirs in healthcare workers, patients, or the hospital environment may be a principle mode of spread in nosocomial outbreaks.

#### Treatment Options

Carbapenemase-producing *K. pneumoniae* strains are currently one of the most important nosocomial pathogens. Hospital outbreaks of KPC-producing *K. pneumoniae* mainly affect severely ill patients and are associated with an increased risk of death (Ducomble et al., 2015; Tumbarello et al., 2015). KPC-producing *K. pneumoniae* bloodstream infections in intensive care unit (ICU) have also been associated with increased mortality (Chang et al., 2015).

Because carbapenemase-producing *K. pneumoniae* are mostly resistant to several important antibiotic classes (β-lactam drugs, fluoroquinolones, and aminoglycosides), antibiotics, such as polymyxin B, colistin (polymyxin E), fosfomycin, tigecycline, and sometimes selected aminoglycosides, are the last-resort agents. KPC-producing *K. pneumoniae* are usually resistant to all β-lactam antibiotics, but temocillin can be active against some KPC-producing *K. pneumoniae*, particularly in the case of lower urinary tract infections (Adams-Haduch et al., 2009). To maximize bacterial killing and minimize bacterial resistance, combined therapy is sometimes recommended. Combination therapy including a carbapenem, such as a combination of tigecycline, colistin, and meropenem, was strongly effective in the treatment of KPC-producing *K. pneumoniae*, including colistin-resistant isolates (Tumbarello et al., 2012, 2015; Giamarellou et al., 2013; Hong et al., 2013; Daikos et al., 2014). The synergistic combination of colistin and rifampin was also effective in the treatment of colistin-resistant KPC-producing *K. pneumoniae* by slowing the selection of heteroresistant subpopulations during colistin therapy (Tascini et al., 2013). However, several reports have shown that combination therapy was not superior to monotherapy (de Oliveira et al., 2015; Toledo et al., 2015). Thus, extensive studies will be required to assess the effectiveness of combination therapy. A triple combination of colistin-doripenem-ertapenem was effective only in isolates with high levels of OmpK35 and OmpK36 porin expression (Hong et al., 2013). The expression level of OmpK36 was also involved in the rapid induction of high-level carbapenem resistance in heteroresistant KPC-producing *K. pneumoniae* populations (Adams-Sapper et al., 2015). Therefore, molecular characterization of the KPC-producing *K. pneumoniae* strain, such as the determination of the expression level of OmpK35 and OmpK36, can be used to identify effective combination regimens. However, as a minor effect of OmpK35 and OmpK36 on carbapenem resistance of *K. pneumoniae* was also reported (Zhang et al., 2014), more extensive studies on the role of these proteins on *K. pneumoniae* carbapenem resistance are also required.

Colistin (polymyxin E), an agent discovered more than 60 years ago, is a key component of the combination of antimicrobial regimens used for the treatment of severe KPC-producing *K. pneumoniae* infections (Cannatelli et al., 2014b). Since the global spread of KPC-producing *K. pneumoniae*, the emergence of colistin resistance in KPC-producing *K. pneumoniae* have been reported in many countries, including Italy (Cannatelli et al., 2014b; Giani et al., 2015), the USA (Bogdanovich et al., 2011), Greece (Kontopoulou et al., 2010), Hungary (Toth et al., 2010), and Turkey (Labarca et al., 2014). The increasing prevalence of colistin-resistant *K. pneumoniae* producing KPC poses a threat to public health because colistin resistance increases the mortality due to KPC-producing *K. pneumoniae* bloodstream infections and reduces therapeutic options. A multicenter case-control-control study in Italy showed that the rate of colistin resistance among KPC-producing *K. pneumoniae* blood isolates increased more than threefold during the 4.5-years study period, and the 30-days mortality due to colistin-resistant KPC-producing *K. pneumoniae* bloodstream infections was approximately 51% (Giacobbe et al., 2015). Data collected from 21 hospital laboratories in Italy between 2013 and 2014 also showed that 43% of carbapenemase-producing *K. pneumoniae* were resistant to colistin, 6% were resistant to tigecycline, 16% were resistant to gentamicin, 82% were resistant to trimethoprim-sulfamethoxazole, and 1% were resistant to all four antibiotics, and colistin-resistant isolates were detected in all participating hospital laboratories (Monaco et al., 2014). The progressive increase of colistin resistance was also reported elsewhere (Pena et al., 2014; Bonura et al., 2015; Parisi et al., 2015). These results indicate that the strict rules for colistin use are required to diminish the spread of colistin resistance in the endemic regions of KPC-producing *K. pneumoniae*.

Molecular and biochemical studies have shown that insertional inactivation of the *mgrB* gene, encoding a negative-feedback regulator of the PhoQ–PhoP signaling system, can be responsible for colistin resistance in KPC-producing *K. pneumoniae*, due to the resulting up-regulation of the Pmr lipopolysaccharide modification system (Cannatelli et al., 2013, 2014b). A recent study analyzing a series of colistin-resistant *K. pneumoniae* isolates of worldwide origin identified a single amino acid change (T157P) in the PmrB protein as being responsible for the overexpression of *pmrCAB* and *pmrHFIJKLM* operons involved in lipopolysaccharide modification, leading to colistin resistance (Jayol et al., 2014). The relationship between colistin resistance and inactivation of the *mgrB* gene was further supported by analysis of clinical colistin-resistant *K. pneumoniae* isolates producing KPC (Bonura et al., 2015; Giani et al., 2015). The emergence of colistin resistance was also associated with low-dosage colistin treatment (Cannatelli et al., 2014a). A recent report showed that the plasmid carrying the *mcr-1* gene, which encodes a phosphoethanolamine transferase enzyme catalyzing the addition of phosphoethanolamine to lipid A, is a major contributor to colistin resistance in Gram-negative bacteria and is spread through horizontal gene transfer (Liu et al., 2016). This *mcr-1*-harboring plasmid was also detected in *E. coli* isolates collected from 78 (15%) of 523 samples of raw meat, 166 (21%) of 804 animals, and 16 (1%) of 1322 samples from inpatients with infection, indicating the emergence of this plasmid-mediated colistin resistance mechanism (Liu et al., 2016).

Fosfomycin is a broad-spectrum antibiotic that inhibits bacterial cell wall biogenesis by inactivating the enzyme UDP-*N*-acetylglucosamine-3-enolpyruvyltransferase, also known as MurA (Brown et al., 1995). Fosfomycin has been used to treat KPC-producing *K. pneumoniae*, but recently, a high fosfomycin resistance rate was observed is in countries with higher usage (Giske, 2015). Only 43.4% of KPC-producing *K. pneumoniae* strains retained susceptibility to fosfomycin in a Chinese university hospital (Li et al., 2012) and a similar fosfomycin susceptibility rate (39.2%) was observed in KPC-producing *K. pneumoniae* collected from 12 hospitals in China (Jiang et al., 2015). Like colistin, fosfomycin resistance seems to be associated with the plasmid containing the *fosA3* gene which encodes glutathione S-transferase to modify fosfomycin and was characterized first in CTX-M-producing *E. coli* in Japan (Wachino et al., 2010). In China, the *fosA3*-harboring plasmid was attributed to 55.6% of fosfomycin-resistant KPC-producing *K. pneumoniae* strains (Jiang et al., 2015). Although the *fosA3* gene is mainly associated with the *bla*_CTX-M_ gene, the *fosA3* gene has also been characterized in atypical *bla*_KPC_-harboring plasmids (Jiang et al., 2015; Li et al., 2015). In pFOS18 (Jiang et al., 2015) and pKP1034 (Xiang et al., 2015), the *fosA3* and *bla*_KPC-2_ genes were located on different transposon systems, whereas in pHS102707 belonging to the IncP1 group (Li et al., 2015), two genes were co-located in the same Tn*1721*-Tn*3*-like transposon.

Tigecycline, a derivative of minocycline, is the first member of the glycylcycline class that acts as a protein synthesis inhibitor by blocking the interaction of aminoacyl-tRNA with the A site of the ribosome (Rose and Rybak, 2006). Due to the increased clinical use of tigecycline for treatment of KPC-producing *K. pneumoniae*, increased tigecycline resistance was reported (Zagorianou et al., 2012; Papadimitriou-Olivgeris et al., 2014; Weterings et al., 2015). In the Netherlands, all KPC-producing *K. pneumoniae* isolates exhibited reduced susceptibility to tigecycline (Weterings et al., 2015). Another report showed that during ICU stay, 17.9% (39/257) of patients became colonized by tigecycline-resistant KPC-producing *K. pneumoniae* during their stay (Papadimitriou-Olivgeris et al., 2014). In a Greek tertiary hospital during 2004 to 2010, 11.3% (34/301) of KPC-producing isolates were resistant to tigecycline (Zagorianou et al., 2012). Overproduction of efflux pumps such as AcrAB and overexpression of RamA, a positive regulator of the AcrAB efflux system, seem to be major factors for decreased sensitivity of *K. pneumoniae* strains to tigecycline (Ruzin et al., 2005; Rosenblum et al., 2011; Sun et al., 2013). A recent report in China showed that the OqxAB efflux pump was also contributed to tigecycline resistance in *K. pneumoniae* isolates (Zhong et al., 2014).

Because KPC-producing *K. pneumoniae* sometimes remains susceptible to several aminoglycosides such as gentamicin (Tzouvelekis et al., 2014), aminoglycosides can be used alone or in combination therapy to treat KPC-producing *K. pneumoniae* infections. Actually, gentamicin monotherapy or in combination with tigecycline was recently reported to reduce the mortality from sepsis caused by *K. pneumoniae* ST512 clone producing KPC-3, SHV-11, or TEM-1 (Gonzalez-Padilla et al., 2015). New weapons are always indispensable for combating KPC-producing *K. pneumoniae* infections (Lee et al., 2007, 2015a). The effectiveness of some antibiotics in development was also estimated against KPC-producing *K. pneumoniae*. Potent inhibitors of serine β-lactamases, such as avibactam and MK7655, were effective against KPC-producing *K. pneumoniae* infections (Temkin et al., 2014). Combination therapy with avibactam and ceftazidime exhibited significant synergetic effects against organisms with combinations of extended-spectrum β-lactamases (ESBLs), AmpCs, and KPC-2 (Wang et al., 2014e). Plazomicin (a novel aminoglycoside) also exhibited significant activity against KPC-producing *K. pneumoniae* (Temkin et al., 2014). The novel polymyxin derivatives with lower nephrotoxicity are under development (Vaara, 2010). A recent report suggested that synthetic peptides with antimicrobial and antibiofilm activities are a promising strategy in the treatment of infections caused by KPC-producing *K. pneumoniae* (Ribeiro et al., 2015). The *in vitro* activity of the next-generation aminoglycoside plazomicin alone and in combination with colistin, meropenem, fosfomycin or tigecycline was tested against carbapenemase-producing *Enterobacteriaceae* (CPE) strains. When plazomicin was combined with meropenem, colistin or fosfomycin, synergy was observed against CPE isolates (Rodriguez-Avial et al., 2015).

#### Detection Methods

Because a delay in the appropriate antibiotic therapy for severe infections of KPC-producing *K. pneumoniae* is strongly associated with unfavorable prognosis and increased mortality rates (Karaiskos and Giamarellou, 2014), rapid detection of CR strains is essential for the effective management of these infections (Lee et al., 2005b, 2006b, 2016). Various methods for the identification of KPCs have been developed, including multiplex PCR assay (Spanu et al., 2012; Adler et al., 2014), real-time PCR assay (Wang et al., 2012; Lee et al., 2015c), DNA microarray (Peter et al., 2012; Braun et al., 2014), Raman spectroscopic analysis (Willemse-Erix et al., 2012), single-colony whole-genome sequencing (Koser et al., 2014), matrix-assisted laser desorption ionization-time-of-flight mass spectrometry (MALDI-TOF MS; Carvalhaes et al., 2014), loop-mediated isothermal amplification (LAMP) method (Nakano et al., 2015), chromogenic medium (Vrioni et al., 2012), and a new phenotypic test, called the carbapenem inactivation method (CIM; van der Zwaluw et al., 2015). False positive results can occur when the modified Hodge test is used to detect carbapenemases in carbapenemase-negative *K. pneumoniae* clinical isolates (Wang et al., 2011). Therefore, to improve the efficiency in the phenotypic detection of KPC-producing *K. pneumoniae* isolates, the modified Hodge test can be combined with an EDTA disk test (Yan et al., 2014) or a disk test using boronic acid compounds (Pournaras et al., 2010a). These methods enhanced the sensitivity and specificity of KPC detection in *K. pneumoniae* isolates. In a new developed phenotypic test, called the CIM, a susceptibility-testing disk containing carbapenem was immersed in the suspension made by suspending an inoculation loop of bacterial culture (van der Zwaluw et al., 2015). After incubation, the disk was placed on an agar plate inoculated with a susceptible *E. coli* indicator strain. If the bacterial isolate produces carbapenemase, the susceptibility-testing disk will allow the growth of the susceptible indicator strain. This method showed high concordance with results obtained by PCR (van der Zwaluw et al., 2015). Nevertheless, these culture-based phenotypic tests are time-consuming and cannot easily detect ESBLs and carbapenemases produced by *Enterobacteriaceae*, owing to varied levels of enzyme expression and the poor specificity of some antibiotic combinations (Okeke et al., 2011; Swayne et al., 2013). To overcome these limitations of phenotypic methods, various molecular-based diagnostic methods have been developed. Especially, the direct detection of the carbapenemase gene using multiplex PCR, real-time PCR, and DNA microarray method can increase the speed and accuracy of detecting CR strains (Okeke et al., 2011; Solanki et al., 2014). Because of the high genetic diversity of genes coding for carbapenemase, the precise design of primers or probes is necessary for correctly amplifying or detecting only expected carbapenemase genes. Therefore, already developed methods for *bla* gene detection are restricted to the detection of only limited types of *bla* genes (Lee et al., 2015b). However, the _large-scale_*bla*Finder was recently developed on the basis of multiplex PCR, and this large-scale detection method can detect almost all *bla* genes, including KPCs, NDMs, OXA-48-likes, present in bacterial pathogens (Lee et al., 2015b). Recently, mass spectrometry-based methods, such as MALDI-TOF MS and ultra-performance liquid chromatography–tandem mass spectrometry (UPLC-MS/MS), have been shown to be capable of characterizing carbapenemase-producing bacteria (Carricajo et al., 2014; Carvalhaes et al., 2014). These methods are fast and accurate to routinely identify bacterial isolates with great specificity and sensitivity (Hrabak et al., 2011; Patel, 2013; Carvalhaes et al., 2014; Lasserre et al., 2015), but these systems do not accurately provide the carbapenem minimum inhibitory concentrations (MICs) for carbapenemase-producing *K. pneumoniae* (Patel, 2013). Several experiments showed that this method is more rapid and accurate for detection of carbapenemase activity in Gram-negative bacteria than some methods including the modified Hodge test (Lee et al., 2013b; Chong et al., 2015). The LAMP method has emerged as a powerful gene amplification assay for the rapid identification of microbial infections (Notomi et al., 2000). This method employs a DNA polymerase and a set of four specially designed primers that recognize a total of six distinct sequences on the target DNA. The assay amplifies the DNA under isothermal conditions (63–65°C) with high degrees of specificity, efficiency, and speed (Reddy et al., 2010). The cycling reaction continues with accumulation of 10^9^ copies of target in less than an hour. The assay can be conducted in a water bath or heating block instead of the thermal cycling using a PCR machine (Notomi et al., 2000). The LAMP assay can be applied for detection of KPC producers in the clinical laboratory (Nakano et al., 2015) and has greater sensitivity, specificity, and rapidity compared to the phenotypic methods and PCR for the detection of KPC-producing *K. pneumoniae* (Solanki et al., 2013).

Colistin has often been used as a therapeutic option for the treatment of CR *K. pneumoniae* infections. However, the imprudent use of colistin has caused rapid spread of colistin resistance in *K. pneumoniae* producing carbapenemases, particularly the KPC-type carbapenemases (Monaco et al., 2014; Giacobbe et al., 2015). This situation demonstrates the need for the development of accurate and reliable methods for detecting colistin resistance. Recently, several methods for the identification of colistin resistance were reported, including various routine colistin MIC testing methods, such as BMD, BMD-P80, AD, Etest, MTS, and Vitek2 (Dafopoulou et al., 2015; Humphries, 2015); capillary electrophoresis method according to characteristic surface properties of bacteria (Sautrey et al., 2015); and the micromax assay based on evaluation of the efficacy of antibiotics that affect cell wall integrity (Tamayo et al., 2013). Because a recent report showed that the *mcr-1* gene, involved in the modification of lipid A, is a major contributor to colistin resistance in Gram-negative bacteria (Liu et al., 2016), detection of this gene may be important in the detection of colistin resistance.

### Class B Carbapenemases

#### Epidemiology

Class B β-lactamases are metallo-β-lactamases that require zinc or another heavy metal for catalysis. Class B β-lactamases have a broad substrate spectrum and can catalyze the hydrolysis of virtually all β-lactam antibiotics including carbapenems except for monobactams (Jeon et al., 2015). Class B carbapenemases were mostly identified in *Enterobacteriaceae* and include VIMs, IMPs, and the emerging NDM group (Jeon et al., 2015). Among them, NDM (New Delhi metallo-β-lactamase) is one of the most clinically significant carbapenemases. NDM-1 was first detected in 2008 in *K. pneumoniae* and *E. coli* in a patient returning to Sweden from India and has since spread worldwide (Yong et al., 2009; Jeon et al., 2015). Thus far, 15 NDM variants have been assigned (Jeon et al., 2015), and most of them originated from Asia (Nordmann and Poirel, 2014). NDMs shares very little identity with other metallo-β-lactamases (Nordmann and Poirel, 2014).

**FIGURE 3:**
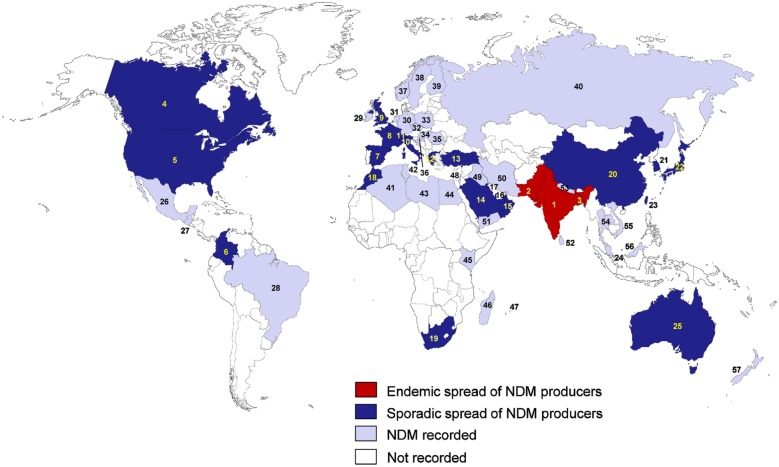
**Epidemiological features of NDM-producing *K. pneumoniae*.** (1) India; (2) Pakistan; (3) Bangladesh; (4) Canada; (5) USA; (6) Colombia; (7) Spain; (8) France; (9) UK; (10) Italy; (11) Switzerland; (12) Greece; (13) Turkey; (14) Saudi Arabia; (15) Oman; (16) United Arab Emirates; (17) Kuwait; (18) Morocco; (19) South Africa; (20) China; (21) South Korea; (22) Japan; (23) Taiwan; (24) Singapore; (25) Australia; (26) Mexico; (27) Guatemala; (28) Brazil; (29) Ireland; (30) Germany; (31) Netherlands; (32) Czech Republic; (33) Poland; (34) Hungary; (35) Romania; (36) Croatia; (37) Norway; (38) Sweden; (39) Finland; (40) Russia; (41) Algeria; (42) Tunisia; (43) Libya; (44) Egypt; (45) Kenya; (46) Madagascar; (47) Mauritius; (48) Israel; (49) Iraq; (50) Iran; (51) Yemen; (52) Sri Lanka; (53) Nepal; (54) Thailand; (55) Vietnam; (56) Malaysia, (57) New Zealand.

Since 2008, *K. pneumoniae* producing NDMs rapidly spread in many countries (Berrazeg et al., 2014; **Figure 3**). NDM-producing *K. pneumoniae* are considered to be endemic in the Indian subcontinent, including India, Pakistan, and Bangladesh (Nordmann et al., 2011b; Nordmann and Poirel, 2014). The sporadic spread has been reported in the USA (Rasheed et al., 2013; Centers for Disease Control and Prevention, 2014; Doi et al., 2014), Canada (Mulvey et al., 2011; Borgia et al., 2012; Lowe et al., 2013), Colombia (Escobar Perez et al., 2013; Ocampo et al., 2015), Spain (Oteo et al., 2013b; Seara et al., 2015), France (Arpin et al., 2012; Robert et al., 2014), Switzerland (Poirel et al., 2011g; Spyropoulou et al., 2016), Italy (Gaibani et al., 2011), the UK (Kumarasamy et al., 2010; Giske et al., 2012), Greece (Voulgari et al., 2014; Spyropoulou et al., 2016), Turkey (Poirel et al., 2014; Kilic and Baysallar, 2015), Morocco (Poirel et al., 2011b; Barguigua et al., 2013), South Africa (Brink et al., 2012; de Jager et al., 2015), Singapore (Chen et al., 2012; Balm et al., 2013; Ling et al., 2015), Saudi Arabia (Shibl et al., 2013; Zowawi et al., 2014), Oman (Poirel et al., 2011a; Zowawi et al., 2014), United Arab Emirates (Sonnevend et al., 2013; Dash et al., 2014), Kuwait (Jamal et al., 2012, 2013; Sonnevend et al., 2015b), China (Qin et al., 2014; Jin et al., 2015; Liu et al., 2015), Japan (Yamamoto et al., 2013; Nakano et al., 2014), Taiwan (Chiu et al., 2013; Tseng et al., 2015), South Korea (Kim et al., 2012; Cho et al., 2015), and Australia (Shoma et al., 2014; Wailan et al., 2015). In India, NDM-1 was the most common carbapenemase type detected and accounted for 75.22% of the carbapenemase-producing isolates (Kazi et al., 2015). In Singapore and the United Arab Emirates, NDM-1 also was the most common carbapenemase type observed (44.4 and 100%, respectively; Dash et al., 2014; Ling et al., 2015). The endemic spread of NDM-producing *K. pneumoniae* has also been reported in the UK, which has close relationships with India and Pakistan (Nordmann and Poirel, 2014). In China, NDM-1 has been found mostly in *Acinetobacter* spp., but data obtained from patients between June 2011 and July 2012 showed that 33.3% of the CRE isolates, including *K. pneumoniae*, had NDM-1, suggesting the possible transmission of *bla*_NDM-1_-containing sequences from *Acinetobacter* spp. to *Enterobacteriaceae* (Qin et al., 2014). These findings reveal the emergence and active transmission of NDM-1-producing *K. pneumoniae* in China. Comparative analyses of the conserved NDM-1-encoding region among different plasmids from *K. pneumoniae* and *E. coli* suggested that the transposable elements and two unknown inverted repeat-associated elements flanking the NDM-1-encoding region aided the spreading of this resistance determinant (Chen et al., 2012). Recently, in China, eight *K. pneumoniae* isolates producing NDM-1 were identified in the neonatal ward of a teaching hospital (Zhang et al., 2015), and four diverse types (NDM-1, KPC-2, VIM-2, and IMP-4) of carbapenemase of *K. pneumoniae* clones were identified in a single hospital in China (Liu et al., 2015).

The Balkan states (Livermore et al., 2011; Voulgari et al., 2014), the Arabian Peninsula (Nordmann and Poirel, 2014), and North African countries (Dortet et al., 2014c), have also been recently considered as an additional reservoir of NDM producers. In the Arabian Peninsula, NDM-1 was the most frequently encountered carbapenemase (46.5%) followed by OXA-48-like carbapenemases (32.5%; Sonnevend et al., 2015b). In Greece, among 132 non-repetitive CRE isolates between 2010 and 2013, 78 *K. pneumoniae* isolates with the *bla*_NDM-1_ gene were identified (Voulgari et al., 2014). In the USA, KPC-producing *K. pneumoniae* have been responsible for much of the increase in carbapenemase-producing bacteria detection, but recent increases in NDM-producing *K. pneumoniae* have the potential to add to this burden (Rasheed et al., 2013; Centers for Disease Control and Prevention, 2014).

The movement of patients between countries may be a trigger for the international spread of carbapenemase-producing *K. pneumoniae* (Berrazeg et al., 2014). Carbapenemase-producing Gram-negative bacteria including *K. pneumoniae*, obtained from patients that had recently traveled outside Canada between 2010 and 2013, were found to be NDM-producing *K. pneumoniae*, belonging to various sequence types (ST15, ST16, ST147, ST258, ST340, ST512, and ST972) with different plasmids (IncF, IncA/C, and IncL/M), and were imported from India to Canada (Peirano et al., 2014). Therefore, more careful attention is required when treating patients with a recent history of foreign hospitalization in countries where carbapenemase-producing bacteria are prevalent. The international transportations of patients between countries recently resulted in the detection of NDM-producing *K. pneumoniae* in Mexico (Barrios et al., 2014), Guatemala (Pasteran et al., 2012), Brazil (Quiles et al., 2015), the Netherlands (Bathoorn et al., 2015), Ireland (McDermott et al., 2012), Poland (Baraniak et al., 2016), Czech Republic (Studentova et al., 2015), Croatia (Kocsis et al., 2016), Russia (Ageevets et al., 2014), Tunisia (Ben Nasr et al., 2013), Romania (Lixandru et al., 2015), Egypt (Bathoorn et al., 2013), Kenya (Poirel et al., 2011f), Madagascar (Chereau et al., 2015), Iraq (Poirel et al., 2011e), Yemen (Gharout-Sait et al., 2014), Iran (Shahcheraghi et al., 2013), Mauritius (Poirel et al., 2012b), Sri Lanka (Dortet et al., 2013), Thailand (Rimrang et al., 2012), Nepal (Tada et al., 2013), Vietnam (Hoang et al., 2013), Malaysia (Al-Marzooq et al., 2015), and New Zealand (Williamson et al., 2012).

The global dissemination of NDM-producing *K. pneumoniae* also has a serious impact on neonatal mortality rates, particularly in low-income countries where the burden of neonatal sepsis is high (Zaidi et al., 2005). Colonization of NDM-producing *K. pneumoniae* isolates in pregnant women in the community in Madagascar highlighted the potential for mother-to-child transmission (Chereau et al., 2015). In India, analysis of *Enterobacteriaceae*, including *K. pneumoniae*, isolated from the blood of septicaemic neonates, indicted that 14% of the isolates possessed *bla*_NDM-1_, and there was a significantly higher incidence of sepsis caused by NDM-1-harboring isolates (Datta et al., 2014). In China, an outbreak of *bla*_NDM-1_-producing *K. pneumoniae* ST20 and ST17 isolates was identified in a neonatal unit (Jin et al., 2015). In Turkey, the spread of NDM-1-producing *K. pneumoniae* in a neonatal ICU was reported (Poirel et al., 2014). In Colombia, NDM-1-producing *K. pneumoniae* strains were identified from an outbreak that affected six neonatal patients (Escobar Perez et al., 2013).

As with KPC, coexistence of NDMs and other carbapenemases in *K. pneumoniae* has also been reported worldwide, in Brazil (NDM-1/KPC-2; Pereira et al., 2015), Malaysia (NDM-1/OXA-232; Al-Marzooq et al., 2015), South Korea (NDM-5/OXA-181; Cho et al., 2015), China (NDM-1/IMP-4; Chen et al., 2015), India (NDM-1/OXA-232; Al-Marzooq et al., 2015), Turkey (NDM-1/OXA-48; Kilic and Baysallar, 2015), Pakistan (NDM-1/KPC-2; Sattar et al., 2014), Switzerland (NDM-1/OXA-48; Seiffert et al., 2014), United Arab Emirates (NDM-1/OXA-48-like; Dash et al., 2014), Australia (NDM-1/OXA-48; Sidjabat et al., 2015), Morocco (NDM-1/OXA-48; Barguigua et al., 2013), Singapore (NDM-1/OXA-181 and NDM-5/OXA-181; Balm et al., 2013), and the USA (NDM-1/OXA-232; Doi et al., 2014).

Besides NDM-type carbapenemases, the IMP and VIM groups have also been detected worldwide in *K. pneumoniae*, but other carbapenemases, such as GIM-1, KHM-1, and SPM-1, have been not found in *K. pneumoniae* (**Table 1**). Since IMP and VIP were first detected in *Serratia marcescens* in 1991 and in *Pseudomonas aeruginosa* in 1996, respectively (Osano et al., 1994; Lauretti et al., 1999), IMP- and VIM-producing *K. pneumoniae* have spread in Europe and Asia, but were rarely reported in other regions, such as America and Africa (**Table 1**). Although in this review we have focused only on carbapenemase-producing *K. pneumoniae*, the VIM group is one of the most commonly reported carbapenemases worldwide if we consider all bacteria species (the VIM groups have been mainly identified in *P. aeruginosa*; Poirel et al., 2007).

#### Molecular and Genetic Context

The *bla*_NDM_ gene is frequently observed in the transposon Tn*125* (with two flanking IS*Aba125* elements) within NDM-producing species of the genus *Acinetobacter* (Partridge and Iredell, 2012; Wailan et al., 2015). The *bla*_NDM_ gene was hypothesized to originate in the genus *Acinetobacter* (Toleman et al., 2012). In *Enterobacteriaceae*, the IS*Aba125* elements of the Tn*125* structure carrying *bla*_NDM_ are frequently truncated (Tn*125*) at various lengths and the Tn*125* structure frequently has different IS elements (**Figure 2B**; Partridge and Iredell, 2012; Wailan et al., 2015). The *bla*_NDM_ genes in *K. pneumoniae* have been reported on numerous broad-host-range plasmid types, including IncA/C (Hudson et al., 2014), IncF (Hishinuma et al., 2013), IncR (Studentova et al., 2015), IncH (Villa et al., 2012), IncN (Chen et al., 2014a), IncL/M (Peirano et al., 2014), and IncX types (Wang et al., 2014d). The predominant plasmid type responsible for spreading *bla*_NDM-1_ is the IncA/C type plasmids (Poirel et al., 2011d; Pitout et al., 2015). Many IncA/C plasmids with *bla*_NDM-1_ also carry various antibiotic resistance genes including 16S rRNA methylases (RmtA and RmtC), associated with aminoglycoside resistance; CMY-type β-lactamases, associated with broad-spectrum cephalosporin resistance; and QnrA, associated with quinolone resistance (Pitout et al., 2015). Consequently, many NDM-producing *K. pneumoniae* were susceptible only to colistin, fosfomycin, and tigecycline (Nordmann and Poirel, 2014). A novel NDM-1 variant (NDM-9) located on a novel IncH variant plasmid was recently identified in a clinical *K. pneumoniae* isolate in China (Wang et al., 2014c). Because the *bla*_NDM_ genes are located in numerous broad-host-range plasmids, the spread of NDM-1 is facilitated by horizontal gene transfer between bacteria. In China, CRE 21 strains harboring the *bla*_NDM-1_ gene were found to consist of multiple *Enterobacteriaceae* species including nine *Enterobacter cloacae*, three *E. coli*, three *Citrobacter freundii*, two *K. pneumoniae*, two *K. oxytoca*, and two *Morganella morganii* strains (Wang et al., 2015).

Unlike KPC, the *bla*_NDM_ genes were detected in various *K. pneumoniae* clones. ST11, a major high-risk sequence type of KPC-producing *K. pneumoniae* in Asia, has also been associated with *bla*_NDM-1_ in *K. pneumoniae* identified in many countries, such as the USA (Rasheed et al., 2013), Greece (Voulgari et al., 2014), Australia (Shoma et al., 2014), Switzerland (Seiffert et al., 2014), the Czech Republic (Studentova et al., 2015), Spain (Oteo et al., 2013b), and Thailand (Netikul et al., 2014). The recent outbreak of NDM-producing *K. pneumoniae* ST11 in Poland was caused by a clone similar to an isolate identified in the Czech Republic in 2013 (Baraniak et al., 2016), indicating the local spread of this clone. ST11 has also been associated with OXA-48-like enzymes from isolates found in Argentina, Turkey, and Spain (Lascols et al., 2013; Oteo et al., 2013b). ST14, ST147, and ST340 have sometimes been associated with *bla*_NDM_ in *K. pneumoniae* in many countries (Peirano et al., 2011, 2014; Poirel et al., 2011d; Giske et al., 2012; Osterblad et al., 2012; Lascols et al., 2013; Gharout-Sait et al., 2014; Lee et al., 2014; Shoma et al., 2014; Wang et al., 2014d; Izdebski et al., 2015; Sonnevend et al., 2015b). Over 50% of NDM-producing *K. pneumoniae* isolates from India belonged to either ST11 or ST147 (Lascols et al., 2013). The analysis of clinical isolates of NDM-1-producing *K. pneumoniae* from India, the UK, and Sweden, showed that the most frequently detected sequence types were ST14, ST11, ST149, ST231, and ST147 (Giske et al., 2012). Although ST258 is a high-risk KPC-producing *K. pneumoniae* clone, ST258 harboring *bla*_NDM_ has never been reported, to the best of our knowledge. This phenomenon may result from the integrative conjugative element ICEKp258.2, present only in ST258 and genetically related sequence types (Adler et al., 2014). This genetic locus contains a type IV pilus gene cluster and a type III restriction-modification system. Because these genes are associated with the restriction of plasmids and specific mobile elements, most ST258 may predominantly harbor plasmids with the *bla*_KPC_ gene. Therefore, PCR for this unique region (ICEKp258.2) can provide a reliable tool for the rapid detection of the ST258 clone.

High-resolution genomic analysis of multidrug-resistant hospital outbreaks of *K. pneumoniae* through whole-genome sequencing revealed the emergence of a capsule switching NDM-1 bearing *K. pneumoniae* ST15 strain, suggesting that further studies should concentrate on the diversity and spread of this specific clone (Chung The et al., 2015). ST15 harboring *bla*_NDM-1_ has often been reported in many countries, including Spain (Ruiz-Garbajosa et al., 2013), Croatia (Kocsis et al., 2016), Thailand (Netikul et al., 2014), Canada (Peirano et al., 2014), China (Hu et al., 2013), France (Arpin et al., 2012), and Morocco (Poirel et al., 2011b). In Bulgaria, this clone was responsible for the clonal dissemination of KPC-2-producing *K. pneumoniae* (Markovska et al., 2015). Another whole-genome sequencing analysis of CR *K. pneumoniae* strains, which were isolated from 26 individuals involved in infections in a Nepali neonatal unit, showed that three temporally separated cases were caused by a single NDM-producing *K. pneumoniae* strain with four conserved plasmids including a plasmid carrying *bla*_NDM-1_ (Stoesser et al., 2014). The plasmids contained a large number of antimicrobial resistance and plasmid maintenance genes, which may explain their persistence (Stoesser et al., 2014). These reports suggest that whole-genome sequencing analysis play an important role in the elucidation of the factors that allow emergence and persistence of resistance.

#### Treatment Options

New-Delhi metallo-β-lactamase-producing *K. pneumoniae* are usually resistant to most β-lactam antibiotics but remain susceptible to aztreonam (Nordmann et al., 2012a). As with the case of KPC-producing *K. pneumoniae*, the effect of combination therapy was tested in the treatment of NDM-producing *K. pneumoniae* infections. When double- and triple-antibiotic combinations of aztreonam, ciprofloxacin, colistin, daptomycin, fosfomycin, meropenem, rifampin, telavancin, tigecycline, and vancomycin were used in patients infected with two NDM-producing *K. pneumoniae* strains susceptible to colistin, the combination of rifampin-meropenem-colistin was the most effective regimen against these strains (Tangden et al., 2014). The *in vitro* synergetic effect of the combination therapy of colistin and fosfomycin against NDM-producing *K. pneumoniae* has also been reported (Bercot et al., 2011). The combination of polymyxin B and chloramphenicol used against NDM-producing *K. pneumoniae* substantially enhanced bacterial killing and suppressed the emergence of polymyxin resistance (Abdul Rahim et al., 2015). Combination therapy including aztreonam and avibactam (a novel inhibitor of serine β-lactamases under development) was effective in the treatment of metallo-β-lactamase-producing bacterial infections (Wang et al., 2014e).

In the case of NDM-1-producing *Enterobacteriaceae* infections, carbapenems have been suggested to still represent a viable treatment option (Wiskirchen et al., 2014b). Despite unfavorable *in vitro* MICs of NDM-producing *K. pneumoniae*, recent *in vivo* studies have demonstrated the efficacy of carbapenems against NDM-1-producing isolates in immunocompetent-mouse and neutropenic-mouse thigh infection models (Wiskirchen et al., 2013, 2014b; MacVane et al., 2014). Although high-dose, prolonged infusions of ertapenem or doripenem induced reduction in bacterial density, bacterial density was also reduced in standard infusions of ertapenem at 1 g every 24 h or of doripenem at 500 mg every 8 h (Wiskirchen et al., 2013, 2014b). Notably, these efficacies were observed only against NDM-1-producing *K. pneumoniae* (Wiskirchen et al., 2013, 2014b). In addition to carbapenems, this discordance between *in vitro* and *in vivo* activities against NDM-1-producing *K. pneumoniae* was also observed in human simulated regimens of ceftazidime at 2 g every 8 h or ceftazidime/avibactam at 2,000/500 mg every 8 h (MacVane et al., 2014). Despite experiments in immunocompetent-mouse and neutropenic-mouse thigh infection models, these results show that standard infusions of ertapenem and doripenem could reduce bacterial density. Therefore, further experiments in human are required to determine whether carbapenems are sometimes a viable treatment option for NDM-1-producing *K. pneumoniae* infections.

The copy number of *bla*_NDM-1_ was assessed using Southern blotting and quantitative PCR under different conditions. The *bla*_NDM-1_ sequence was maintained under antibiotic selection; however, removal of the antibiotic selection led to the emergence of susceptible bacterial populations with a reduced copy number or even the complete loss of the *bla*_NDM-1_ gene (Huang et al., 2013). The dynamic nature of the copy number of *bla*_NDM-1_ provides a strong argument for the prudent use of clinically important antibiotics to reduce the development and dissemination of antibiotic resistance among pathogenic bacteria (Huang et al., 2013).

#### Detection Methods

Because NDM-producing *K. pneumoniae* infections are also associated with significant in-hospital mortality (de Jager et al., 2015), the rapid and accurate detection of NDM-producing *K. pneumoniae* is becoming a major issue in limiting the spread of CR bacteria. Several methods recently developed to detect NDM-producing *K. pneumoniae* include Xpert^®^ Carba-R based on real time PCR (Anandan et al., 2015), the Carba NP test based on the color change of a pH indicator (Nordmann et al., 2012b), and its derivatives (Pires et al., 2013; Dortet et al., 2014b), and a method based on MALDI-TOF (Hrabak et al., 2013a). Xpert^®^ Carba-R based on real time PCR effectively identified various carbapenemases of KPC, NDM, IMP, and VIM, with 100% sensitivity and 77% specificity (Anandan et al., 2015). However, this method failed to detect OXA-48-like carbapenemases, in contrast to multiplex PCR (Anandan et al., 2015).

The Carba NP test using chromogenic medium is based on the color change of a pH indicator (Nordmann et al., 2012b). The enzymatic hydrolysis of the β-lactam ring of a carbapenem (usually imipenem) causes the acidification of an indicator solution (phenol red for the Carba NP test) that changes its color due to pH modification (Garcia-Fernandez et al., 2016). This method could rapidly detect KPC, IMP, VIM, NDM, and OXA-48-like producers with sensitivity and specificity of 97.9 and 100%, respectively, directly from spiked blood cultures (Dortet et al., 2014a). Comparative evaluation of the Carba NP test with other detection methods was tested in many reports (Vasoo et al., 2013; Huang et al., 2014; Yusuf et al., 2014; Gallagher et al., 2015; Lifshitz et al., 2016). When the Carba NP test and the modified Hodge test were compared, the Carba NP test was more specific (100% versus 80%) and faster (Vasoo et al., 2013; Huang et al., 2014; Yusuf et al., 2014). When it was compared the performance of the Carba NP test and the commercially available imipenem hydrolysis-based rapid test (the Rosco Rapid CARB screen kit) for detecting CPE and *P. aeruginosa*, the Carba NP test showed superior specificity and sensitivity (Huang et al., 2014; Yusuf et al., 2014; Gallagher et al., 2015). A novel simplified protocol of the Carba NP test designed for carbapenemase detection direct from bacterial cultures (instead of bacterial extracts) showed enhanced detection of carbapenemase producers (Pasteran et al., 2015).

However, several reports showed that false-negative results in the Carba NP test were associated with mucoid strains or linked to enzymes with low carbapenemase activity, particularly OXA-48-like (Tijet et al., 2013; Osterblad et al., 2014). To overcome these problems, several derivatives of the Carba NP test were developed, such as the Rapidec Carba NP test (bioMérieux; Poirel and Nordmann, 2015), the CarbAcineto NP test for rapid detection of carbapenemase-producing *Acinetobacter* spp. (Dortet et al., 2014b), the Rapid CARB Screen (Rosco Diagnostica; Dortet et al., 2015), the Blue-Carba test using bromothymol blue as a pH indicator solution (Garcia-Fernandez et al., 2016), a modified Carba NP test (Bakour et al., 2015a), and the BYG Carba test based on an electro-active polymer biosensing technology (Bogaerts et al., 2016). Many studies evaluated the performance of the Rapidec Carba NP test (bioMérieux), which was introduced into the market for the detection of carbapenemase production (Dortet et al., 2015; Hombach et al., 2015; Kabir et al., 2016; Lifshitz et al., 2016). These report showed that this method was user-friendly and had a high overall performance, making it an attractive option for clinical laboratories (Kabir et al., 2016). Recently, performance evaluation of two biochemical rapid tests commercialized (the Rapidec Carba NP test and the Rapid CARB Screen) was reported and compared with the home-made Carba NP test (Dortet et al., 2015). The Rapidec CARBA NP test possesses the best performance for rapid and efficient detection of CPE (Dortet et al., 2015). The BYG Carba test based on a new and original electrochemical method detects the variations of conductivity of a polyaniline (an electro-sensing polymer)-coated electrode which is highly sensitive to the modifications of pH and of redox activity occurring during the imipenem enzymatic hydrolysis reaction (Bogaerts et al., 2016). In comparison with PCR results, the BYG Carba test displayed sensitivity of 95% and specificity of 100% versus 89% and 100%, respectively, for the Carba NP test (Bogaerts et al., 2016). The development of these detection methods based on inexpensive and affordable techniques can limit the spread of CR bacteria.

### Class D Carbapenemases

#### Epidemiology

**FIGURE 4:**
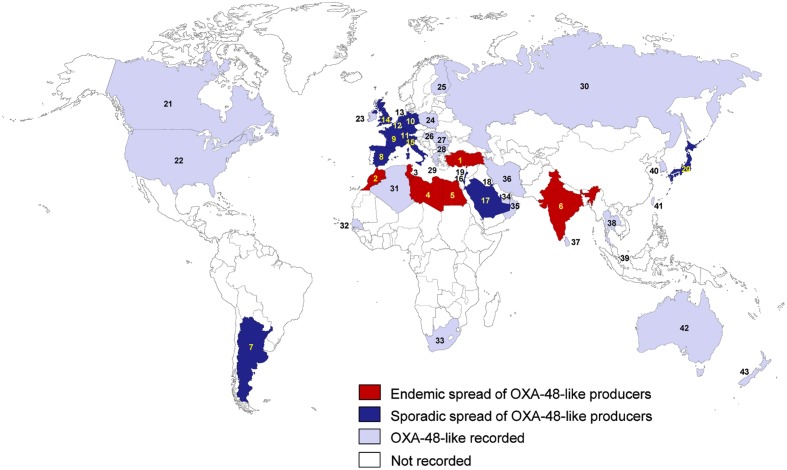
**Epidemiological features of OXA-48-like-producing *K. pneumoniae*.** (1) Turkey; (2) Morocco; (3) Tunisia; (4) Libya; (5) Egypt; (6) India; (7) Argentina; (8) Spain; (9) France; (10) Germany; (11) Switzerland; (12) Belgium; (13) Netherlands; (14) UK; (15) Italy; (16) Israel; (17) Saudi Arabia; (18) Kuwait; (19) Lebanon; (20) Japan; (21) Canada; (22) USA; (23) Ireland; (24) Poland; (25) Finland; (26) Hungary; (27) Romania; (28) Bulgaria; (29) Greece; (30) Russia; (31) Algeria; (32) Senegal; (33) South Africa; (34) United Arab Emirates; (35) Oman; (36) Iran; (37) Sri Lanka; (38) Thailand; (39) Singapore; (40) South Korea; (41) Taiwan; (42) Australia; (43) New Zealand.

Class D β-lactamases were referred to as oxacillinases (OXAs) because they commonly hydrolyze isoxazolylpenicillins (oxacillin, cloxacillin, and dicloxacillin) much faster than benzylpenicillin (Jeon et al., 2015). Of over 400 Class D β-lactamases, only some variants actually possess carbapenemase activity. Based on their amino acid sequence, class D carbapenemases were recently reclassified into 12 subgroups: OXA-23, OXA-24/40, OXA-48, OXA-51, OXA-58, OXA-134a, OXA-143, OXA-211, OXA-213, OXA-214, OXA-229, and OXA-235 (Jeon et al., 2015). Among them, only several subgroups such as OXA-23, OXA-48, OXA-51, and OXA-58 are reported in *K. pneumoniae* (Evans and Amyes, 2014). OXA-48 is the most efficient class D carbapenemase for imipenem and is one of the most prevalent class D carbapenemases (Jeon et al., 2015). The OXA-48 was first identified in *K. pneumoniae* in Turkey in 2003 (Poirel et al., 2004), and thus far, 10 variants of the *bla*_OXA-48_ gene have been identified (Jeon et al., 2015). Turkey may be one of the main reservoirs of OXA-48-producing *K. pneumoniae* (Nordmann and Poirel, 2014). Since 2003, the endemic spread of these bacteria has been reported in countries such as Turkey, Morocco, Libya, Egypt, Tunisia, and India (Nordmann and Poirel, 2014; **Figure 4**). The sporadic spread has been reported in France (Liapis et al., 2014; Semin-Pelletier et al., 2015), Spain (Oteo et al., 2013b; Pena et al., 2014), Italy (Giani et al., 2013, 2014), Belgium (Cuzon et al., 2008; Huang et al., 2013), the Netherlands (Kalpoe et al., 2011; Dautzenberg et al., 2014), the UK (Dimou et al., 2012; Thomas et al., 2013), Germany (Pfeifer et al., 2012; Kola et al., 2015), Switzerland (Potron et al., 2012; Seiffert et al., 2014), Argentina (Poirel et al., 2011c; Lascols et al., 2013), Lebanon (Beyrouthy et al., 2014), Israel (Adler et al., 2013, 2015), Kuwait (Poirel et al., 2012a; Zowawi et al., 2014), Saudi Arabia (Shibl et al., 2013; Liu et al., 2015), and Japan (Nagano et al., 2013; Hashimoto et al., 2014). The prevalence of OXA-48 carbapenemases among carbapenemase-producing *K. pneumoniae* in Spain and France was particularly high (74 and 78%, respectively; Robert et al., 2014; Palacios-Baena et al., 2016). In Africa, OXA-48-producing *K. pneumoniae* have been mainly reported in the northern countries, such as Morocco, Libya, Egypt, Tunisia, and Algeria (**Figure 4**). In the Arabian Peninsula, the prevalence of OXA-48-like carbapenemases among carbapenemase-producing *K. pneumoniae* was also high (32.5–56%; Zowawi et al., 2014; Sonnevend et al., 2015b). Among CRE isolates in Lebanon, 88% produced OXA-48 carbapenemase (Beyrouthy et al., 2014). In Saudi Arabia, 78% of carbapenemase-producing *K. pneumoniae* isolates harbored *bla*_OXA-48_, and three strains of 47 *bla*_OXA-48_-positive *K. pneumoniae* isolates were resistant to colistin, suggesting that colistin resistance is emerging in Saudi Arabia (Shibl et al., 2013).

In France, examination of the epidemiologic features of an outbreak of OXA-48-producing *K. pneumoniae* in an ICU revealed that the outbreak was caused by environmental persistence of OXA-48-producing *K. pneumoniae* over 20 months (Pantel et al., 2016). This report emphasizes the importance of early environmental screening to interrupt the transmission of carbapenemase-producing *K. pneumoniae* (Pantel et al., 2016). Similarly, a large outbreak of OXA-48 carbapenemase-producing *K. pneumoniae* in a French university hospital was recently attributed to the late implementation of successive cohort units and a high level of staff movement between the infectious diseases and internal medicine ward (Semin-Pelletier et al., 2015). These results suggest that practical guidelines are required to help hospitals confronting uncontrolled outbreaks. Because the gut of colonized patients is the main source of CPE, accurate and stringent hygiene of endoscopic instruments is also important. A recent report partially attributed an outbreak of OXA-48-producing *K. pneumoniae* in a German University hospital to complex instruments such as the duodenoscope (Kola et al., 2015). Strict hygiene regulations for various nosocomial environments, including endoscopic instruments as well as gowns and gloves, are required.

OXA-48-producing *K. pneumoniae* was recently also detected in Canada (Ellis et al., 2013), the USA (Mathers et al., 2013), Ireland (Wrenn et al., 2014), Poland (Izdebski et al., 2015), Hungary (Janvari et al., 2014), Greece (Voulgari et al., 2013), Romania (Lixandru et al., 2015), Bulgaria (Markovska et al., 2015), Finland (Osterblad et al., 2012), Russia (Fursova et al., 2015), Algeria (Cuzon et al., 2015), United Arab Emirates (Ahn et al., 2015), Iran (Azimi et al., 2014), South Africa (Brink et al., 2013), Senegal (Moquet et al., 2011), Taiwan (Ma et al., 2015), Singapore (Ling et al., 2015), South Korea (Jeong et al., 2015), and Australia (Espedido et al., 2013; **Figure 4**). In North America, the frequency of OXA-48-like enzymes among carbapenemase-producing *K. pneumoniae* isolates was very low (11%; Lascols et al., 2013). A recent report in Romania showed that among 65 carbapenemase-producing *K. pneumoniae*, the most frequently identified gene was the *bla*_OXA-48_ gene (78%), 12% were positive for *bla*_NDM-1_ gene, 6% had the *bla*_KPC-2_ gene (Lixandru et al., 2015). The recent spread of OXA-48 and OXA-244 carbapenemase genes in Russia was reported among *Proteus mirabilis*, *E. aerogenes*, and *E. cloacae* as well as *K. pneumoniae* (Fursova et al., 2015). In South Africa, the emergence of a colistin-resistant OXA-181-producing *K. pneumoniae* isolate was also reported (Brink et al., 2013), and in the Netherlands, an OXA-48-producing *K. pneumoniae* was reported to infect two patients (Kalpoe et al., 2011), and a hospital-wide outbreak was successfully controlled (Dautzenberg et al., 2014).

OXA-181, a derivative of OXA48 with the substitution of a single amino acid, was first identified in India in Potron et al. (2011), and then has been spread to many different countries, such as the UK (Dimou et al., 2012), Romania (Székely et al., 2013), Canada (Peirano et al., 2014), Oman (Potron et al., 2011), Singapore (Balm et al., 2013), Sri Lanka (Hall et al., 2014), South Korea (Cho et al., 2015), Australia (Sidjabat et al., 2015), Japan (Kayama et al., 2015), and New Zealand (Williamson et al., 2011). However, in many cases, the infections have been associated with India (Potron et al., 2011; Williamson et al., 2011; Dimou et al., 2012; Hall et al., 2014; Peirano et al., 2014). Other OXA-48-derivatives, such as OXA-204 (Potron et al., 2013), OXA-232 (Potron et al., 2013), and OXA-163 (Poirel et al., 2011b), were recently identified in Tunisia, France, and Argentina, respectively, and OXA-244 and OXA-245 were first reported in Spain (Oteo et al., 2013a). All of these countries are regions with a high prevalence of OXA-48-producing *K. pneumoniae* (**Figure 4**). In addition, OXA-232-producing *K. pneumoniae* has been reported in various countries, such as the USA (Doi et al., 2014), Singapore (Teo et al., 2013), India (Al-Marzooq et al., 2015), and South Korea (Jeong et al., 2015). These results indicate that besides OXA-48, its many derivatives also spread worldwide. OXA-163, which differs from OXA-48 by a four amino acid deletion and a single amino acid substitution, has lower carbapenemase activity than OXA-48, but this enzyme exhibits extended activity against expanded-spectrum cephalosporins, and its activity is partially inhibited by clavulanic acid, a β-lactamase inhibitor (Poirel et al., 2011c). OXA-247, a derivative of OXA-163 with a single amino acid substitution, was recently reported in Argentina (Gomez et al., 2013), where OXA-163-producing *K. pneumoniae* were often reported (Lascols et al., 2013).

The coexistence of OXA-48-like and other carbapenemases in *K. pneumoniae* was also frequently reported worldwide, such as in Turkey (OXA-48/NDM-1; Kilic and Baysallar, 2015), Switzerland (OXA-48/NDM-1; Seiffert et al., 2014), United Arab Emirates (OXA-48-like/NDM-1; Dash et al., 2014), Australia (OXA-48/ NDM-1; Sidjabat et al., 2015), Morocco (OXA-48/NDM-1; Barguigua et al., 2013), India (OXA-181/VIM-5; Castanheira et al., 2011), Singapore (OXA-181/NDM-1 and OXA-181/NDM-5; Balm et al., 2013), the USA (OXA-232/NDM-1; Doi et al., 2014), and India (OXA-232/NDM-1; Al-Marzooq et al., 2015).

Other class D carbapenemases, such as OXA-23, OXA-24/40, OXA-51, OXA-58, OXA-134a, OXA-143, OXA-211, OXA-213, OXA-214, OXA-229, and OXA-235, were mainly identified in *Acinetobacter* species such as *A. baumannii* but not in *K. pneumoniae* (**Table 1**; Evans and Amyes, 2014).

#### Molecular and Genetic Context

Unlike KPCs and NDMs, one highly transferable IncL group plasmid (pOXA-48a) was mainly responsible for the spread of the *bla*_OXA-48_ gene in *K. pneumoniae* (**Figure 2C**; Pitout et al., 2015). The molecular epidemiology of OXA-48 in European and North African countries showed that in 92.5% of the isolates, the *bla*_OXA-48_ gene was located on this self-conjugative IncL/M type plasmid (Potron et al., 2013). The *bla*_OXA-48_ gene was found only in the IncL group of IncL/M type plasmids (Carattoli et al., 2015). In contrast to the IncA/C plasmids of NDM-1, the pOXA-48a plasmid contains *bla*_OXA-48_, a unique antibiotic resistance gene (Pitout et al., 2015). The conjugation rate of the pOXA-48a plasmid was very high (1 × 10^-1^); therefore, this self-conjugative plasmid can conjugate at a very high frequency to *Enterobacteriaceae* (Potron et al., 2014). Inactivation of the *tir* gene, which is known to encode a transfer inhibition protein, was recently reported to be responsible for a 50- to 100-fold increase in the efficiency of transfer of the pOXA-48a plasmid (Potron et al., 2014), explaining the spread of the pOXA-48a plasmid with *bla*_OXA-48_. Recently, the *bla*_OXA-48-like_ gene has also been reported on other plasmids and genetic elements, such as IncA/C types (Ma et al., 2015), IncH types (Wang et al., 2014c), and Tn*1999* (Poirel et al., 2012a). In comparison to the pOXA-48a plasmid, the transmission frequency of the Tn*1999* composite transposon was very low (<1.0 × 10^-7^; Aubert et al., 2006).

OXA-48 has most often been found in *K. pneumoniae*, but OXA-48 was also found in various *Enterobacteriaceae*, because of the high conjugation rate of the pOXA-48a (Potron et al., 2014). Molecular and epidemiological analyses in a German hospital showed the horizontal gene transfer of the OXA-48-containing plasmid from *K. pneumoniae* to *E. coli* (Gottig et al., 2015). Besides *E. coli*, OXA-48 has also been identified in *K. oxytoca*, *Enterobacter* spp., *Providencia rettgeri*, *C. freundii*, and *S. marcescens* (Poirel et al., 2012c; Berger et al., 2013). The *bla*_OXA-48_ gene was identified in all *Enterobacteriaceae* isolates from the index case in Spain, indicating the capacity of OXA-48 carbapenemase to spread among *Enterobacteriaceae* by the horizontal gene transfer (Arana et al., 2015).

Similar to NDM, the *bla*_OXA-48-like_ genes were detected in various *K. pneumoniae* clones. ST11 has often been associated with *bla*_OXA-48-like_ in *K. pneumoniae* isolated in many countries, such as Spain (Oteo et al., 2013a,b, 2015; Ruiz-Garbajosa et al., 2013; Branas et al., 2015), Taiwan (Ma et al., 2015), Libya (Lafeuille et al., 2013), Turkey (Lascols et al., 2013), Argentina (Lascols et al., 2013), and Greece (Voulgari et al., 2013). In Spain, a large outbreak was initiated in 2013 by a OXA-48-producing *K. pneumoniae* ST11 clone, and this strain was detected in 44 patients (Branas et al., 2015). The ST11 isolates carried various carbapenemases, including NDM-1, VIM-1, OXA-48, KPC-2, and OXA-245 (Lascols et al., 2013; Oteo et al., 2013b, 2015). In addition, ST14, ST15, ST101, ST147, and ST405 harboring *bla*_OXA-48-like_ have often been reported in many countries, such as the USA (Lascols et al., 2013), Spain (Oteo et al., 2013b, 2015; Ruiz-Garbajosa et al., 2013; Arana et al., 2015; Cubero et al., 2015), the Czech Republic (Hrabak et al., 2015), Libya (Lafeuille et al., 2013), India (Lascols et al., 2013), Germany (Gottig et al., 2015), Finland (Osterblad et al., 2012), France (Liapis et al., 2014), and Japan (Hashimoto et al., 2014). Recent results from 83 hospitals in Spain showed that OXA-48 (71.5%) and VIM-1 (25.3%) were the most frequently detected carbapenemases, and the most prevalent sequence types were ST11 and ST405 for *K. pneumoniae* (Oteo et al., 2015). However, the molecular epidemiology of OXA-48-producing enterobacterial isolates collected from European and north-African countries between 2001 and 2011 indicated that ST101 was the most commonly observed sequence type in *K. pneumoniae* isolates, accounting for 17 out of 67 isolates (25.4%), followed by ST395 and ST15 (each seven isolates, 10.5%; Potron et al., 2013). Two outbreaks of OXA-48-producing *K. pneumoniae* ST101 clones were reported in Spain (Pitart et al., 2011; Cubero et al., 2015). OXA-48-producing *K. pneumoniae* appear to vary geographically. As with NDM, to the best of our knowledge, ST258 harboring *bla*_OXA-48-like_ has never been reported.

#### Treatment Options

OXA-48-like-producing *K. pneumoniae* are usually resistant to most β-lactam antibiotics, but OXA-48-producing *K. pneumoniae* without ESBLs remain susceptible to the expanded-spectrum cephalosporins (Nordmann et al., 2012a; Munoz-Price et al., 2013). In addition, OXA-48-like-producing *K. pneumoniae* sometimes remains susceptible to several aminoglycosides such as gentamicin (Tzouvelekis et al., 2014). Unlike for NDMs (Wiskirchen et al., 2013, 2014b; MacVane et al., 2014), carbapenems may not be a reliable treatment option for OXA-48 producer infection (Wiskirchen et al., 2014a). Combination therapy with sulbactam, meropenem, and colistin was more effective in isolates producing NDM carbapenemase than those producing OXA-48-like carbapenemases, suggesting that the identification of the carbapenemase type helps determine the combination most likely to clear the infection (Laishram et al., 2015). The combination of fosfomycin with imipenem, meropenem, and tigecycline was also synergistic against OXA 48-positive *K. pneumoniae* strains *in vitro* with the ratios of 42, 33, and 33%, respectively (Evren et al., 2013). Similarly, the *in vitro* assays indicate that imipenem-containing combinations were effective against serine-β-lactamase producers (KPC, OXA-48), while no synergy was observed for all NDM-1 producers (Poirel et al., 2016). However, because carbapenems were effective against NDM-1-producing isolates *in vivo* (Wiskirchen et al., 2013, 2014a; MacVane et al., 2014), the effect of carbapenem combination therapy on carbapenemase-producing isolates should be determined *in vivo*, particularly in the case of NDM-1-producing bacteria.

Despite the effectiveness of combination therapies, the prognosis for bloodstream infections caused by OXA-48-producing *Enterobacteriaceae* remains poor, and the 30-days mortality reached 50% (Navarro-San Francisco et al., 2013). A similar result was reported in OXA-48-producing *K. pneumoniae* infections in a tertiary hospital in Spain (Pano-Pardo et al., 2013). Although OXA-48-producing *K. pneumoniae* were susceptible to several antibiotics, including amikacin (97.2% susceptible), colistin (90.1%), tigecycline (73%), and fosfomycin (66.2%), in-hospital mortality among patients with OXA-48-producing *K. pneumoniae* infections was 43.5% (Pano-Pardo et al., 2013). Therefore, to prevent delay in diagnosis and initiation of optimal antimicrobial therapy, rapid identification of OXA-48-producing isolates is required.

#### Detection Methods

As with KPCs and NDMs, various detection methods were developed to identify OXA-48-like carbapenemases (Tsakris et al., 2010; Giske et al., 2011; Girlich et al., 2013; Naas et al., 2013; Lee et al., 2015c). Accurate differentiation of the various carbapenemase types, such as KPC-type, NDM-type, and OXA-48-type enzymes, is crucial for controlling the spread of carbapenem resistance among *Enterobacteriaceae* (Nordmann et al., 2012a). Many phenotypic detection methods to allow differentiation between class A and class B carbapenemases were developed using boronic acid derivatives and EDTA or dipicolinic acid (Tsakris et al., 2010; Giske et al., 2011). Recently, a specific phenotypic method to differentiate a single OXA-48 producer from those producing other carbapenemase types (e.g., KPC-types, NDM-types) was also developed (Tsakris et al., 2015). This method was based on an imipenem disk and two blank disks adjacent to the imipenem disk, loaded with the tested strain and impregnated with EDTA and EDTA plus phenyl boronic acid, respectively (Tsakris et al., 2015). This novel method exhibited 96.3% sensitivity and 97.7% specificity (Tsakris et al., 2015). ChromID OXA-48, based on chromogenic media, have been commercialized for the direct isolation of CPE from clinical samples (Girlich et al., 2013), with an estimated 91% sensitivity and 100% specificity (Girlich et al., 2013).

### Emerging Class C Carbapenemases

Class C β-lactamases confer resistance to penicillins, cephalosporins, and cephamycins (cefoxitin and cefotetan) and are not significantly inhibited by clinically applied β-lactamase inhibitors such as clavulanic acid (Jeon et al., 2015). Although four class C carbapenemases (ACT-1, CMY-2, CMY-10, and ADC-68) have been reported, ACT-1 and CMY-2 exhibit reduced susceptibility to carbapenems, particularly ertapenem, only when combined with permeability defects, due to their low catalytic efficiencies (*K_cat_*/*K_m_*) for imipenem (0.007 and 0.04 M^-1^⋅S^-1^, respectively; Mammeri et al., 2010). CMY-10 with the catalytic efficiency of 0.14 M^-1^⋅S^-1^ for imipenem was the first reported carbapenemase among plasmidic class C β-lactamases (Kim et al., 2006), and this enzyme was also a class C extended-spectrum β-lactamase with extended substrate specificity for extended-spectrum cephalosporins (Lee et al., 2009, 2012a; Jeon et al., 2015). Among the chromosomal class C β-lactamases, ADC-68 identified in *A. baumannii* was the first reported enzyme possessing both class C extended-spectrum β-lactamase and carbapenemase activities (Jeon et al., 2014, 2015), and its catalytic efficiency for imipenem is 0.17 M^-1^ S^-1^. CMY-10-producing *K. pneumoniae* was identified only in South Korea (Lee et al., 2005a, 2006a) and ADC-68-producing *K. pneumoniae* was never reported.

Many reports showed that carbapenem resistance can be triggered by the loss of two major porins, OmpK35 and OmpK36, in combination with ESBLs or Ambler class C AmpC cephalosporinases, and the production of carbapenemase (Wang et al., 2009; Shin et al., 2012; Tsai et al., 2013). A recently genetically engineered mutant of *K. pneumoniae* showed that several carbapenems (imipenem, meropenem, and doripenem) remain effective against these carbapenemase-independent CR strains (Tsai et al., 2013). Therefore, laboratory testing for susceptibility to imipenem, meropenem, and doripenem can improve the accuracy of identification of these isolates (Tsai et al., 2013).

## Conclusion

We analyzed the epidemiology of *K. pneumoniae* producing true carbapenemases (Ambler molecular class A, B, D, and several carbapenemases of class C) responsible for non-susceptibility to carbapenems without additional permeability defects. Many types of CR *K. pneumoniae* have been identified worldwide (**Figures 1**, **3**, and **4**). During the past 3 years, many countries have reported the arrival of carbapenemases previously unreported in those countries. For example, although *K. pneumoniae* producing KPC- and NDM-type carbapenemases have been extensively reported in the USA, a *K. pneumoniae* producing OXA-48-type carbapenemases was recently detected in the USA (Mathers et al., 2013). Since a first report of NDM-1 in 2008, this carbapenemase has rapidly spread worldwide, and NDM-producing *K. pneumoniae* still continues to be found in new countries, implying that NDM-producing *K. pneumoniae* is still spreading quickly. Despite the global dissemination of KPC, NDM, and OXA-48, the prevalence of carbapenemases varies geographically. The frequency of KPC- and NDM-producing strains was significantly higher in the USA, Canada, Greece, Taiwan, Colombia, and China, whereas OXA-48-producing strains were rarely found in those countries (**Figures 1**, **3**, and **4**). In Argentina, despite the extensive spread of KPC- and OXA-48-producing strains, no NDM-producing *K. pneumoniae* has been reported. Although NDM- and OXA-48-producing *K. pneumoniae* significantly spread in Turkey, KPC-producing strains have rarely been reported there. In Brazil, KPC-producing *K. pneumoniae* has been mainly reported. In the Arabian Peninsula, OXA-48 and NDM producers are common, whereas KPC-type, VIM-type, or IMP-type producers are rare. In India, Spain, France, Italy, and the UK, all three types of carbapenemases have been frequently reported (**Figures 1**, **3**, and **4**).

ST258 is an important strain responsible for the extensive global spread of KPC-producing *K. pneumoniae*. Although the precise reason for the predominance of the ST258 strain in KPC-producing *K. pneumoniae* is not fully understood, recent molecular studies unveiled the genetic characteristics of this strain. The ST258 strains consists of two distinct genetic clades (*cps*-1 and *cps*-2) derived from genetic differentiation in genes involved in capsule polysaccharide biosynthesis. In addition, the integrative conjugative element ICEKp258.2, present only in ST258 and genetically related sequence types, may be linked to the global success of the ST258 clone. ICEKp258.2 contains a type IV pilus gene cluster and a type III restriction-modification system. The type IV pilus gene cluster ICEKp258.2, particularly *pilV*, may contribute to the global success of ST258 clone. The type III restriction-modification system associated with the restriction of plasmids and specific mobile elements may explain the differences observed between ST258 predominantly harboring KPC and ST11, another high-risk clone that lacks ICEKp258.2, harboring various carbapenemases, such as NDM-1, OXA-48, KPC-2, VIM-1, and OXA-245. IncF with FII_K_ replicons, a plasmid most commonly identified in ST258 with *bla*_KPC_, often contains several genes associated with resistance to other antibiotics, such as aminoglycosides, tetracyclines, quinolones, trimethoprim, and sulfonamides. The features of this plasmid may also play an important role in the current global success of ST258.

The rapid global spread of NDM-type carbapenemases may be partly attributed to the dissemination of various epidemic broad-host-range plasmids bearing the *bla*_NDM_ genes. NDM-type carbapenemases were found in various plasmids such as IncA/C, IncF, IncR, IncH, IncN, IncL/M, and IncX types. The IncA/C type plasmids, most common plasmids associated with spread of the *bla*_NDM_ genes, often have various antibiotic resistance genes, such as 16S rRNA methylases associated with aminoglycoside resistance, CMY-type β-lactamases associated with broad-spectrum cephalosporin resistance, and QnrA associated with quinolone resistance. These features may be linked to the current global success of NDM-producing *K. pneumoniae*.

The current spread of OXA-48-producing bacteria is attributed to the pOX-48a plasmid, which belongs to the IncL group of IncL/M type plasmids. Although the pOXA-48a plasmid contains *bla*_OXA-48_, a unique antibiotic resistance gene, the conjugation rate of the pOXA-48a plasmid was very high, which may be responsible for its global spread in *K. pneumoniae*. The high pOXA-48a conjugation rate was recently attributed to mutations in the *tir* gene known to encode a transfer inhibition protein, which may lead to a 50- to 100-fold increase in the efficiency of transfer of the pOXA-48a plasmid. Furthermore, the pOXA-48a plasmid is self-conjugative. Therefore, these specific features of the pOXA-48a plasmid may explain the global dissemination of OXA-48-type carbapenemases.

ST11, ST14, ST101, ST147, and ST258 are major carbapenemase-producing *K. pneumoniae* clones. ST258 was mainly found in KPC-producing *K. pneumoniae*, whereas other clones were found in various carbapenemase-producing *K. pneumoniae* regardless of carbapenemase types. Well-designed epidemiological and molecular studies will be required to understand the dynamics of transmission, risk factors, and reservoirs of these *K. pneumoniae* clones. This will provide information essential for preventing infections and the spread of these risky sequence types.

Most currently available antibiotics may be not sufficiently effective for the treatment of all types of carbapenemase producers in monotherapy. Combination therapy of carbapenems with polymyxin B, colistin, rifampin, fosfomycin, or tigecycline has been reported to effectively treat carbapenemase-producing *K. pneumoniae*. Despite these data supporting the use of combination therapy for treatment of severe carbapenemase-producing *K. pneumoniae* infections, current clinical evidence for treatment guidelines are limited and more accurate randomized controlled *in vivo* studies are required. Moreover, considerable caution is required when applying these therapies. For example, temocillin can actively treat against some KPC-producing *K. pneumoniae*, particularly lower urinary tract infections, and NDM-producing *K. pneumoniae* is often susceptible to aztreonam. OXA-48-producing *K. pneumoniae* remain susceptible to the expanded-spectrum cephalosporins in approximately 20% of cases without ESBLs. In the case of NDM-1-producing *K. pneumoniae*, carbapenems were recently reported to represent a viable treatment option for infections caused by these bacteria, despite unfavorable *in vitro* MICs. Because these results imply that carbapenems can sometimes be a viable treatment option for infection with carbapenemase producers, more extensive studies on the effect of carbapenem monotherapy will be required in the case of NDM-producing *K. pneumoniae* infections.

The accurate and rapid detection of the genotype of carbapenemases can minimize the delay to appropriate prescription of antibiotics. Many detection kits based on various phenotypic or molecular techniques, such as multiplex PCR assay, real-time PCR assay, DNA microarray, Raman spectroscopic analysis, single-colony whole-genome sequencing, MALDI-TOF MS, loop-mediated isothermal amplification method, chromogenic medium, and new phenotypic test methods, have been developed. Through the imprudent use of colistin which is a key component used for the treatment of severe carbapenemase-producing *K. pneumoniae* infections, the rapid spread of colistin resistance was recently reported in *K. pneumoniae* producing carbapenemases, particularly KPC-type carbapenemases. This situation strongly demonstrates the need for the development of novel accurate and reliable methods for detecting resistance to clinically important antimicrobial agents, such as colistin. Hospital interventions can effectively reduce the spread of carbapenemase-producing *K. pneumoniae*. Standard infection control guidelines should be implemented upon the detection of carbapenemase-producing *K. pneumoniae*, and carbapenemase-producing *K. pneumoniae* positive patients should be individually isolated and treated according to strict standard guidelines.

## Author Contributions

C-RL, JL, and SL contributed to the conception and the design of the review and C-RL, JL, KP, YK, BJ, and SL researched and wrote the review.

## Conflict of Interest Statement

The authors declare that the research was conducted in the absence of any commercial or financial relationships that could be construed as a potential conflict of interest.
